# ORDB, HORDE, ODORactor and other on-line knowledge resources of olfactory receptor-odorant interactions

**DOI:** 10.1093/database/baw132

**Published:** 2016-10-01

**Authors:** Luis Marenco, Rixin Wang, Robert McDougal, Tsviya Olender, Michal Twik, Elspeth Bruford, Xinyi Liu, Jian Zhang, Doron Lancet, Gordon Shepherd, Chiquito Crasto

**Affiliations:** 1 Department of Neuroscience; 2 Yale Center for Medical Informatics, Yale University School of Medicine, New Haven, CT, USA; 3 Department of Molecular Genetics, Weizmann Institute of Science, Rehovot, 76100, Israel; 4 HUGO Gene Nomenclature Committee, European Molecular Biology Laboratory, European Bioinformatics Institute, Wellcome Genome Campus, Hinxton, Cambridgeshire CB10 1SD, UK; 5 Department of Pathophysiology, Key Laboratory of Cell Differentiation and Apoptosis of Chinese Ministry of Education, Shanghai JiaoTong University, School of Medicine, Shanghai, China; 6 Center for Biotechnology and Genomics, Texas Tech University, Lubbock, TX, USA

## Abstract

We present here an exploration of the evolution of three well-established, web-based resources dedicated to the dissemination of information related to olfactory receptors (ORs) and their functional ligands, odorants. These resources are: the Olfactory Receptor Database (ORDB), the Human Olfactory Data Explorer (HORDE) and ODORactor. ORDB is a repository of genomic and proteomic information related to ORs and other chemosensory receptors, such as taste and pheromone receptors. Three companion databases closely integrated with ORDB are OdorDB, ORModelDB and OdorMapDB; these resources are part of the SenseLab suite of databases (http://senselab.med.yale.edu). HORDE (http://genome.weizmann.ac.il/horde/) is a semi-automatically populated database of the OR repertoires of human and several mammals. ODORactor (http://mdl.shsmu.edu.cn/ODORactor/) provides information related to OR-odorant interactions from the perspective of the odorant. All three resources are connected to each other via web-links.

**Database URL**: http://senselab.med.yale.edu; http://genome.weizmann.ac.il/horde/; http://mdl.shsmu.edu.cn/ODORactor/

## Introduction

Olfactory receptors (ORs) play a crucial role in the perception of olfaction. An OR interacts with an odorant (or a complex combination of odorants—that represents an odor) from the external environment. This interaction catalyses the sequence of events that culminate in the olfactory processing region of the brain—and, possibly, beyond—leading to the perception of smell. The discovery of the first OR gene was reported in 1991 (1) and earned its discoverers, Linda Buck and Richard Axel, the Nobel Prize in Medicine in 2004 (2). This, and the impetus provided by the recognition that olfactory receptors constitute the largest superfamily of mammalian genomes (3–9), spurred a burgeoning research effort in domains, ranging from the neurosciences to clinical applications.

Several challenges, however, hamper our understanding of the mechanisms underlying the role of ORs in olfaction. One of these concerns is how the discrimination of tens of thousands of odorants, (combined into odors) is facilitated by a few hundred receptors. Consider the recent discovery of nearly 5000 ORs identified within the elephant genome—approximately 2000 of which are functional (10). This is more than twice the number of functional ORs identified from the mouse, rat or dog genomes. The human genome contains far fewer [than 400 (4, 6, 8, 11)] functional ORs. All of these ORs have to be cataloged and archived as they are discovered; and, the information related to the ORs then has to be disseminated to the community rapidly and efficaciously.

OR super families in mammalian genomes are potentially rich repositories of knowledge, especially when associated with rational panels of odorants that activate or inhibit the encoded ORs. Experiments in heterologous expression of OR genes and deorphanizing ORs are however, beset by challenges. This restricts the number of ORs expressed, and consequently, available for functional analysis. The mechanism of the promiscuous OR-odorant interactions (one OR may bind and be activated by multiple odorants; odorants, in turn, bind and activate more than one OR) is also not well understood (12). Consequently, the costs of identifying a focused panel of odorants to test OR responses are prohibitive.

An experimentally determined structure of an OR using x-ray crystallography or solution (e.g. Nuclear Magnetic Resonance) methods is also not yet available. There have been significant efforts in the last decade and a half to provide a mechanistic basis for OR-odorant interactions at a molecular level through computational methods (13–19) These methods have involved creating OR protein models and simulating interactions with odorants using static or dynamic methods. Modeling studies are critical because they provide a mechanistic view of OR-odorant interactions at a molecular level. Different methodologies have been adopted for creating OR models. The structures that arise from these, potentially competing, modeling strategies have to be shared. An additional challenge is that of working towards a uniform and universal nomenclature for ORs.

The development and evolution of the freely web-accessible olfaction resources, SenseLab, HORDE and ODORactor, represent significant efforts towards addressing the issues mentioned in the Introduction.

## Olfactory databases in SenseLab: ORDB, OdorDB, ORMapDB and ORModelDB

### Database schema

The olfaction-related databases in SenseLab, Olfactory Receptor Database (ORDB), OdorDB, ORMapDB and ORModelDB use the EAV/CR (**E**ntity **A**ttribute **V**alue with **C**lasses and **R**elationships) database-schema (20). Such a schema facilitates the efficacious and rapid addition of new information (and, indeed, even creating new databases within SenseLab) and curating and updating existing information remotely (via the Internet) without the need for additional software and database development (21). This schema has facilitated the automatic population and domain evaluation of SenseLab’s olfactory databases for nearly two decades (22). A video of the use of ORDB, OdorDB, ORModelDB and a multi-parameter search feature for all three resources can be found at: https://www.youtube.com/watch?v=MSR_9AQzK2o

All SenseLab olfactory databases can also be accessed through the Neuroscience Information Framework (http://neuinfo.org) (23, 24), a dynamic and federated scientific data aggregator which focuses on disseminating content dedicated to the neurosciences.

Table 1 summarizes the information that is stored and disseminated in the SenseLab databases, ORDB, OdorDB, ORModelDB and ORMapDB—especially as it relates to the Entity-Attribute-Value paradigm: the number of entries and a list of attributes or descriptors for each database.

**Table 1:** The table depicts a summary of the four olfaction-related databases in SenseLab

Database	ORDB	OdorDB	OdorMapDB	ORModelDB
Entries	18 717	257	48	8
**Attribute list**	Name	Name	Name	Name
	Description	Description	Description	Description
	Receptor Type	Chem. Abstract.	Species^a^	GenBank Link^b^
	Organism^a^	Number	Strain	Species^a^
	Source Tissue	Molecular.	Sex	PubMed^b^
	Chromosome	Formula	Age	Receptor Name
	GenBank Link^b^	Functional Group	Weight	Ligands
	PubMed Link^c^	Cyclical Nature	Brain Region	Functional Anal. Ref^b^
	Strain	Hydrocarbon	Sub Region	Modeling Ref^b^.
	Common Name	Feature	Side	Model 3-D structure
	Gene Source	2D Model	View	PDB-formatted Link
	Sequence Lab.	Glomerular	Serial Number	
	Length	Activity Map	Odor	
	Ligand		Odorant Blend	
	Nucleotide Sequence		Concentration	
	Amino Acid Sequence		Exposure Time	
	Sequence Type		Exposure Frequency	
	EXPASY Link		Method	
	Molecular Models		Note	
	Functional Anal.		Authors	
	Lab		PubMed Link^b^	
	Functional Anal. Ref.^b^		Map Picture	
	Microarray		Scan Number Control	
	Experiment.		Scan Number	
			Stimulate	
			Subject Count	
			Image Size	
			Primary Data	
			Behavioral Study^b^	

The table shows the number of entries for each information as well as a list of the attributes or descriptors for each entry in each database. The EAV/CR data-base architecture also allows the storage of information available in different databases stored in common tables in the databases. ‘a’ depicts Organism or Species Name; ‘b’ depicts a link to an entry in GenBank; ‘c’ depicts a link to the biomedical literature in PubMed. The underlined attributes for each database are linked to one or more of the other four databases.

### ORDB

ORDB (http://senselab.med.yale.edu/ORDB) aids users in accessing genomics and proteomics information related to olfactory and other chemosensory receptors: fungal pulmonary receptors, insect ORs, taste papilla receptors, pheromone receptors and vomeronasal receptors. Table 1 lists the attributes for entries in ORDB.

Due in large part to mining the olfactory repertoires of genomes (3–8, 25–27), and aided by semi-automated (22) and remote population via web-based forms (28, 29), and the recent addition of ORs from the African elephant genome, ORDB entries number >18 000. Entries that are not user-supplied have been mined from 6500 GenBank records. ORDB entries represent research that is described in >450 scientific articles. The efforts of the laboratories of >125 researchers are represented. ORDB houses chemosensory receptors from >70 organisms and 35 different source tissues.

Following the publications of the OR repertoires for human and mouse (3, 4, 6, 8, 27), comprehensive sequence analyses were undertaken to identify and catalog similar sequences identified by different groups. These sequences are categorized on the basis of chromosomal location. Color codes are used to identify full length, partial, pseudogene and partial-pseudogene sequences. References to OR sequences whose chromosomal locations are disputed are differently colored. The results of these analyses are available for human (http://senselab.med.yale.edu/ORDB/info/humanorseqanal.htm) and mouse (http://senselab.med.yale.edu/ORDB/info/mouseorseqanal.htm). Each entry in the human OR sequence analysis page is linked to its sources in the HORDE database and the Human Genome GPCR Discovery OR database. A mirror site for the latter is housed within SenseLab. The information contained in these two web pages is updated to reflect modifications to the human and mouse olfactory repertoires.

### OdorDB

OdorDB (http://senselab.med.yale.edu/odordb) was designed to reflect the functional analysis of ORs, namely to identify odorants hat interacted with ORs. In OdorDB, odorant can also be queried against the ORs that they are known to activate via the link, http://senselab.med.yale.edu/odorDB/Summary?cl=22&at=82. Clicking on the link for a receptor takes the user to the entry for that receptor in ORDB.

OdorDB currently lists 257 odorants, 75 of which are known to activate ORs.

### ORModelDB

ORModelDB (http://senselab.med.yale.edu/ORModelDB) catalogs the results of computational studies associated with ORs. This newest olfaction database in SenseLab responds to a need to elucidate the mechanisms of OR-odorant interactions at a molecular level, especially because of a lack of an experimentally derived structure of an OR. This in turns necessitates the use of *ab initio* or semi-empirical methods to create computational models of ORs.(13, 14, 16–19, 30, 31) ORModelDB entries are the results of studies which involve creating computational models of ORs, OR-odorant docking and simulations of OR-odorant interactions

For every OR model entry, the user can also view and manipulate the OR model structure in three dimensions with an embedded viewer powered by the JavaScript library, JSmol (32). The JSmol viewer allows users to manipulate the OR-model views via animation, zooming, surfaces, secondary structures and also measures distances and angles within the OR protein structure.

ORModelDB currently stores eight models. This sparsity is not due to a lack of effort in populating the database; rather, it reflects the research efforts in computational model development for chemosensory receptors. This, in turn, is a reflection of the fact that a very small percentage or ORs have been experimentally, functionally assessed—for reasons previously stated in this paper. The population of ORModelDB proceeds in the same manner as for most of the other olfaction-related databases. A user (with privileges) can access a form that contains text-fields which allow a user to enter information. The form also prompts the user to upload pictures movie files and a PDB file. Once the PDB file is uploaded, the JavaScript automatically invokes the 3D model viewer. The PDB formatted file for an OR model is available for sharing. Model sharing is critical for the model-building effort, especially because model-building protocols by different research groups are procedurally different (13–17, 19, 33).

### OdorMapDB

OdorMapDB is a web resource that presents information related to the molecular and functional organization of the olfactory bulb from 2-deoxyglucose, c-fos and high resolution fMRI experiments. The primary information that OdorMapDB provides is related to maps of the olfactory bulbs (34). These provide comparisons between activity patterns that result from odorant stimulation in the glomerular layer by the above-mentioned experimental methods. OdorMapDB contains 48 entries and includes links to ORDB and OdorDB.

### Integration of ORDB, OdorDB, OdorMapDB and ORModelDB

**Figure 1:**
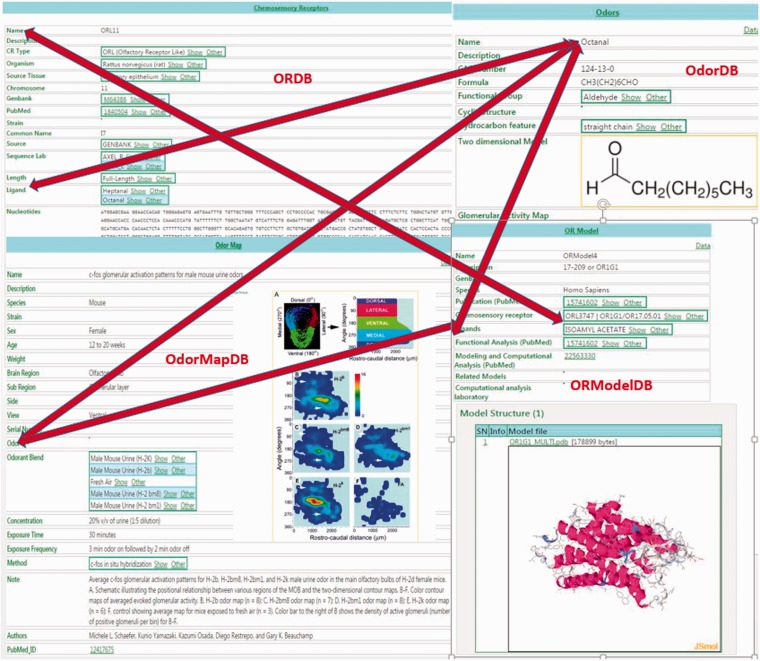
The figure illustrates the integration between the four olfaction databases in SenseLab: ORDB, OdorDB, OdorMapDB and ORModelDB. The scans of entries in each of the databases are highlighted. The double-headed arrows show the integration among the resources. The ‘Ligand’ attribute (see Table 1 for attributes) in ORDB ‘octanal’ can be accessed in OdorDB, as can the ‘Ligand’ attribute in ORModelDB, as well as the ‘Odor’ attribute in OdorMapDB. The ‘Chemosensory Receptor’ attribute in ORModelDB can be accessed through ORDB. The EAV/CR architecture allows the storage of information without the need for replication. Similarly, the OR name can be accessed from OdorDB as well as ORModelDB.

Because ORDB, OdorDB, OdorMapDB and ORModelDB are designed on the same EAV/CR schema (20, 21), easy integration is possible between and among them. Figure 1 illustrates their integration: accessing any one of the four databases in turn allows access to the other three databases.

In ORDB, the ‘ligand’ attribute contains the names of odorant molecules that have been experimentally shown to interact with and activate ORs. Clicking on this ligand-value link lets the user access the page for that ligand-odorant in OdorDB. Alternatively, OdorDB allows users to access a list of receptors that have been identified as being associated with an ‘odorant’ listed in OdorMapDB. The ‘model’ attribute in ORDB provides a link to the model for an OR in OrModelDB. In ORModelDB two attributes for an OR model, ‘chemosensory receptor’ and ‘ligand’, allow access to ORDB and OdorDB, respectively. In ORMapDB the ‘odorant’ attribute links to that odorant in OdorDB.

### Multiple parameter searching in SenseLab

The integration and uniformity of the SenseLab platform facilitates not only the building and incorporation of new databases into SenseLab, but also facilitates the addition of new features. If a new feature is added to a database it automatically becomes available for incorporation into the other databases. One example of such a feature is ‘multiple parameter searching’. This feature is invoked by clicking on the ‘Search’ link in any of the databases; the link for the search in ORDB is https://senselab.med.yale.edu/ORDB/Search. Developing this feature became necessary when ORDB entries increased more than 10-fold after receptor repertoires from genome-wide sequence analyses of ORs (referred to earlier) became available.

An *ad hoc*, multi-parameter search is critical for datasets with a large number of parameters and entries, and mitigates the need to scroll through several pages, or have prior knowledge of the information contained within pages before the relevant entry is found. Because information for each entity in all the databases is organized and described using attributes, the parameters that narrow a search are ‘attributes’ for each entity in the databases (35). For example, a user who knows the name of an entry can search only by typing a few characters of the name of that entry. If a user uses the ‘chromosome’ attribute as the search parameter and enters the chromosome number, all entries related to that chromosome will be returned. Every attribute for an entry can be used simultaneously to focus the search result. For example, a search filter for Organism = Rattus Norvegicus, Chromosome = 11 and Sequence_Lab = Buck_L (Linda Buck) will produce a single link to the ORL11 entry in ORDB.

It is not necessary to know the binomial name for a species or the abbreviated form in which the name of a laboratory leader is stored. For an attribute that is used as a search parameter, if the corresponding values are stored as entities a ‘LookUp’ auto-complete window appears as a link. ‘Search’ also support partial matches: if a user enters ‘%’ before a search-string, e.g. ‘%cow’, the same search following a query of ‘Bos taurus’ will be executed. Part of the video on YouTube (https://www.youtube.com/watch?v=MSR_9AQzK2o) demonstrates the use of multi-parameter searching. Search results can be obtained as HTML (a link to the entry or entries), text and XML (e**X**tensible **M**arkup **L**anguage). Due to the dynamic nature of the EAV/CR storage approach, this search interface automatically adapts to changes in the database structure and content. For example, if a new attribute to better describe an OR, odorant or OR model is added it can immediately be used as a filter in the search software.

## HORDE

The **H**uman **O**lfactory **R**eceptor **D**ata **E**xplorer (genome.weizmann.ac.il/horde/) presents a complete compendium of human OR genes, including pseudogenes and segregating pseudogenes, i.e. genes that have both an intact and disrupted allele in the population. HORDE is also a comprehensive representation of OR orthologs from six other mammalian species: chimpanzee, dog, opossum, platypus and most recently cow, mouse and rat. HORDE is populated by an automated computational pipeline which mines the relevant genes out of complete genomes (36). The pipeline also has the capacity to annotate OR pseudogenes, whose genomic identification is non-trivial. Further, HORDE’s automatic pipeline generates gene symbols based on a sequence similarity classification for each gene (37). For example, the *OR1A2* gene nomenclature utilizes the root OR and indicates the gene is ‘OR family 1 subfamily A member 2’. This nomenclature system has subsequently been applied to dog (9), opossum (38), platypus (39), cow, mouse and rat. Its potential as a tool for the study of mammalian ecological adaptation was recently portrayed (40).

HORDE integrates diverse bioinformatics analyses and additional resources into the database, in an attempt to provide insights into the evolution, structure and function of the complete human OR repertoire. Thus, information is provided on the genomic organization of the ORs into clusters, expression data (obtained from ESTs and microarray data), gene models, annotation of putative functional amino-acid residues, and identification of syntenic clusters (36). This information is pertinent to several open questions such as the control of gene expression in the olfactory system, including epithelial zone-specific expression as well as expression with locus and allele exclusion, ectopic expression in non-olfactory tissues, such as sperm, and biased expression of certain OR genes and pseudogenes.

Genetic variations in OR genes underlie odorant sensitivity differences, specific anosmias (diminished sensitivity) and specific hyperosmias (enhanced sensitivity) (41). To address the universe of human inter-individual variation, the HORDE system integrates a comprehensive catalog of genomic variations in human ORs, collected from 11 sources (42) including the 1000 Genomes Project (43, 44). This encompasses single nucleotide polymorphisms and copy number variation, as well as deleterious mutational events. Using this catalog, HORDE presents a wide range of inferred protein variants (haplotypes) for all intact OR genes, potentially related to differences in odorant binding. The catalog provides a faithful view of the personal OR repertoire, where about two thirds of the loci segregate between intact and inactivated alleles, and every individual genome contains different combinations of allelic ORs.

A major update in HORDE involved the recent addition of mouse and rat ORs to this database. HORDE now contains an updated repertoire of mouse ORs based on the mm10 genome version [GCA_000001635.2]. An integrated set of OR genes obtained from the MGI and NCBI Gene databases were used as input to the HORDE pipeline (36). The resultant mouse OR repertoire contains 1386 genes including 246 pseudogenes, consistent with a recent report (10). HORDE’s pipeline, when applied to the rat genome (version rn6), resulted in a complete and non-redundant list of 1790 rat OR genes including 465 pseudogenes.

**Figure 2:**
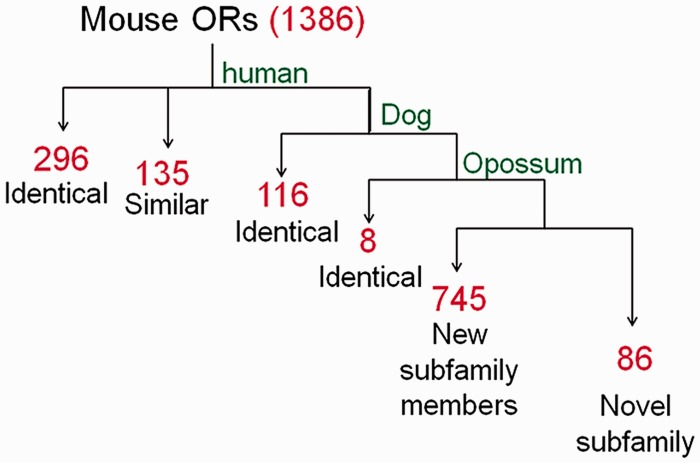
The nomenclature symbol assignment process applied to mouse OR genes. Using the MMS algorithm we compared the mouse OR repertoire to human, dog and opossum and performed a hierarchical symbol assignment as described in the text. Identical: genes identified as mutual-best-hit; similar: genes identified as additional ortholog candidates (second-, third-best, etc.). Red numerals indicate the count of gene symbols assigned to genes in each of the pipeline steps.

For symbol assignment in HORDE the Mutual Maximum Similarity (MMS) algorithm, a systematic classifier, is used to assign a human-based nomenclature to any OR gene based on the hierarchical similarity relationships between the two species. The MMS algorithm analyses the all versus all human-mouse FASTA identity matrix, strives to maximize the symbol similarity between the species, and assigns symbols. Thus, mutual-best-hits are first identified and assigned the symbol of the human best match gene, provided that their similarity is >80%. At a second stage, the algorithm searches for additional orthology candidates with highest similarity to their human partner (≥80%), by allowing more than a single ortholog. Analogous to human ORs mouse OR symbols, such as *Or10aa4-ps*, use the OR root and indicate the gene is ‘OR family10 subfamily aa member 4’, with the optional ‘ps’ suffix to indicate a pseudogene. The symbols for candidate co-orthologs are, e.g. *Or10d4*, *Or10d4b* and *Or10d4c*, with *Or10d4* being the mutual-best-hit. The MMS algorithm was used to compare the mouse and rat OR repertoires to human, as well as dog and opossum, and assigned a symbol for each rodent gene (Figure 2). For mouse 31% of the symbols assigned were identical to human symbols, reflecting orthology. An additional 63% of the symbols were classified into pre-defined OR subfamilies. The remainder (6%) were classified into novel OR subfamilies. In rat, 86% of the genes were assigned the same symbol as their mouse ortholog. The nomenclature was further supported by synteny and phylogenetic analyses. The mouse and rat genes have now been incorporated into HORDE.

HORDE has evolved this method of systematic naming which has identified species-specific expansions in mouse, rat and human, demonstrating the power of this unified nomenclature in generating a framework for studying mammalian OR evolution. The nomenclature can be expanded to serve as a basis for analysing ORs in other mammals. A video detailing HORDE’s usage can be found at: https://www.youtube.com/watch?v=XpiKDCK97jk. Wherever relevant, links to ORDB and OdorDB have also been created.

## ODORactor

**Figure 3:**
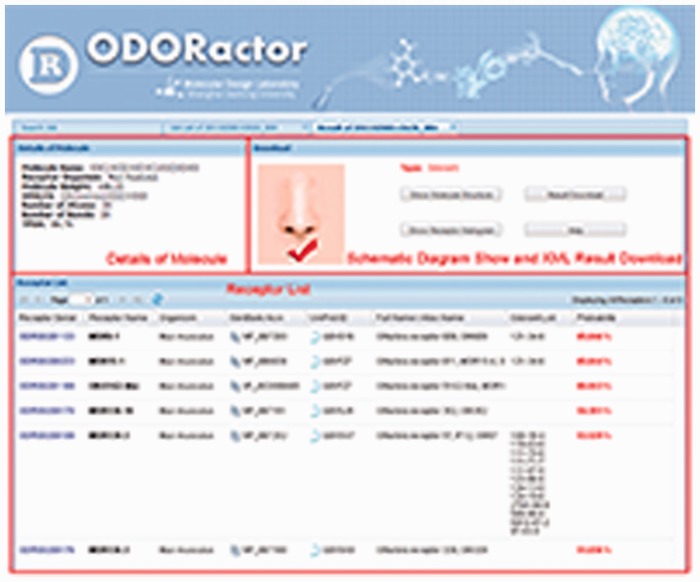
ODORactor results that demonstrate probabilities for interaction between an organic molecule queried (using the SMILES input format) and mouse ORs. The figure shows that there are six possible receptors that are likely to bind the organic molecule with probabilities ranging from 85 to 51%. Links to the receptors in GenBank, UniProt and other olfactory databases are also indicated.

‘ODORactor’ approaches the notion of OR-odorant interactions from the perspective of small molecule compounds, each a putative odorant. Users of ODORactor can query the resource using an organic molecule to identify ORs that are likely to bind an odorant using four formats. SMILES (**S**implified **M**olecular **I**nput **L**ine **E**ntry **S**pecification) is a simplified format that involves the user typing the molecular structure in a single line (Figure 3) Although this format does not provide information related to the two- or 3D structure of the compound, it does identify connectivities between atoms in the molecules. The user can also input the putative odorant molecule by uploading the structure of the molecule in the MDL .mol file format. The .mol file contains information about atoms, bonds, connectivities and coordinates of the atoms in the structure. ODORactor also allows users to sketch the molecule within the web-page, using a JAVA enabled sketching program called MarvinSketch. Efforts are currently underway to replace the JAVA program with a javascript plugin which will simply the molecule sketching program. Users can also access the software by entering the Chemical Abstracts Service identifier for the molecule (45).

The ODORactor server presents novel approaches towards identifying and predicting ORs that might be activated (or inhibited) by small molecule compounds. To accomplish this, two independent models are employed: the first is a classification model for odorants, in which a query (putative odorant) molecule is associated with one or more of over 300 physicochemical descriptors, and used to train a support vector machine (SVM) model. This is followed by another probability-based classification model for receptor recognition, in which both physicochemical descriptors on odorants and PseAAC (pseudo amino acid composition) descriptors on proteins are combined to train another SVM model. Tests have revealed that the model for odorant determination shows a high accuracy of ∼95%, with ∼87% accuracy for receptor recognition.

To evaluate the performance of ODORactor in predicting OR recognition, a fragment library composed of 210 small organic compounds was screened and evaluated for their olfactory responses in mouse. Three organic compounds were predicted as odorants and their potential receptors were computationally recognized. Furthermore, all the interactions between three compounds and the top five corresponding receptors identified via the ODORactor were confirmed from the results of experimental studies, and seven ORs among the predicted candidates were validated to bind the compounds. This predictor is an effective way to decipher olfactory coding and could be a useful server tool for both basic olfactory research in academia and odorant discovery in industry.

In addition, ODORactor features a comprehensive repertoire of olfactory information that matches deorphanized receptors to their corresponding ligands. The ODORactor web server is freely available at http://mdl.shsmu.edu.cn/ODORactor/. Step by step instructions on how to use Odoractor can be accessed at: http://mdl.shsmu.edu.cn/ODORactor/module/mainpage/help.jsp#example0

## Discussion

### Resource interactivity

To serve the community effectively, there is a need for these stable, well-established and often-visited resources to interact with each other. To allow synergisms between the presented resources, and to facilitate their use, olfaction resources in SenseLab, HORDE and ODORactor are interlinked. This ensures that the comprehensibility of information-dissemination is maintained.

*SenseLab and HORDE.* In the comparative sequence analysis of human ORs page at https://senselab.med.yale.edu/ORDB/info/humanorseqanal, all OR contributions from HORDE are linked back to the relevant pages for those OR within HORDE. For example, in the sequence analysis page (this gene name can be found by doing a text search within that web page) the OR that bears the HORDE gene symbol OR10K1 has the link http://genome.weizmann.ac.il/horde/card/index/symbol:OR10K1 to the page in HORDE that contains information for that OR. The HORDE information includes ‘External Ids’, which in turn are links to this OR in different databases. For example, the HORDE OR10K1 receptor has the ORDB name ORL3001 with the corresponding ORDB link https://senselab.med.yale.edu/ORDB/Data/14669.

*SenseLab and ODORactor.* The browse feature in ODORactor (http://mdl.shsmu.edu.cn/ODORactor/module/browse/browse.jsp) contains two tabs, each containing a tabulated list of receptors and odorants, which are pre-stored in the ODORactor databases. ODORactor contains a comprehensive lists of putative odorants; the odorants in OdorDB are a subset of this list. The odorants stored in OdorDB are primarily those that have been known to interact with ORs. Users can browse the tables of receptors and odorants in OdorActor. Each of these tables contains separate columns containing links to ORDB (for receptors) and OdorDB (for odorants).

### Exhaustiveness of information contained

Every resource mentioned in this work is an exhaustive representation, specific to the domain of information contained therein. This is facilitated due to the curators’ efforts in developing and deploying software that populates these resources in automated and semi-automated ways. Every OR gene that is currently resides in Genbank is now in ORDB. More than twice the genes mined from GenBank have been supplied by ORDB users. Information for most of these genes are not publicly accessible anywhere else. SenseLab curators are constantly contacting researchers with requests to submit their gene information after a relevant paper has been published. This is in addition to scouring resources of OR gene information. The HORDE database taps into mammalian (especially primate) genomes as they are published and, for comparison, uses a comprehensive internal and external compendia of ORs (ORDB being one source) to identify OR repertoires. OdorDB is not designed to be a comprehensive repository of every organic compound (a putative odorant), but focuses on a subset of compounds that are likely to have interacted with ORs. The curators of ODORactor, on the other hand, have chosen to be such a comprehensive repository—with a view to the future when a significantly larger number of ORs has been deorphanized. ODORactor stores >3000 odors. ORModelDB, though sparsely populated does indeed represent the nature of this sub-domain of OR research. Because of the significant effort that goes into using semi-empirical (often conflicting methodologies) to design computational OR models, OR models are few; but, the resource still represents >80% of the models that have been created. OdorMapDB represents a novel methodology to process activity patterns following stimulation with odors. As of the writing of this paper, ORMapDB contains information for all such experiments.

### Usage

The olfaction resources in SenseLab, HORDE as ODORactor are well established and stable resources. Each resource has continually evolved in keeping with the evolution in the domain of study. All the resources described in this article continue to remain popular. In order to quantify this popularity, curators of SenseLab, HORDE and ODORactor have complied usage statistics, summaries of which are available online. SenseLab, in the last few years, has used Google Analytics to determine not only the hits (which are often through search engine queries), but also the unique visits for each page and also the bandwidth associated with the hits and visits. The usage statistics for SenseLab’s olfaction databases can be found at: https://senselab.med.yale.edu/OrDB/info/ordb_stats. This link contains information processed from Google Analytics results for 2015. These statistics will be compiled and published every year.

Usage statistics for ODORactor are described by a series bar diagrams that depicts the resource’s use from 2011 [when ODORactor was first created (45) to date. This information is included in the help pages and can be accessed at: http://mdl.shsmu.edu.cn/ODORactor/module/mainpage/help.jsp#stat0.

HORDE curators also use Google Analytics to compile usage statistics. These are updated every six months and usage summaries can be accessed within the link for the HORDE user guide at: http://genome.weizmann.ac.il/horde/pages/user_guide.

## Conclusions

Each of the above described databases is freely accessible and available through the World Wide Web. ORDB represents the field of chemoreception from the perspective of the receptors. The HORDE resource focuses on enhancing our understanding of the olfactory repertoires of mammals and utilizes sequence comparisons to provide standardized gene nomenclature that reflects OR evolution. OdorDB and OdorMapDB represents aspects of the functional responses in the central olfactory processing region of the brain that results from interactions with an OR, while ORModelDB entries provides a glimpse of these interactions at the molecular and structural levels. ODORactor, a comprehensive compendium of odorants, represents a targeted effort towards meeting the challenges of elucidating OR-odorant interactions.

Each of the resources described in this article has been developed independently. Each provides links to a wealth of other resources containing information pertinent to chemosensory receptors and/or odorants. Navigating between these resources enables users to access a plethora of data that can enhance their understanding of ORs and odorants.

## Funding

This work was supported by: (grant numbers R21DC011068, R01 DC009977, UL1 RR024139, T15 LM007056 and U41 HG003345) from the National Institutes of Health.

*Conflict of interest*. None declared.
